# When a Stone Tries to Climb up a Slope: The Interplay between Lexical and Perceptual Animacy in Referential Choices

**DOI:** 10.3389/fpsyg.2013.00154

**Published:** 2013-04-01

**Authors:** Jorrig Vogels, Emiel Krahmer, Alfons Maes

**Affiliations:** ^1^Tilburg center for Cognition and Communication, Tilburg UniversityTilburg, Netherlands

**Keywords:** perceptual animacy, lexical animacy, referring expressions, conceptual accessibility, story retelling, Dutch

## Abstract

Several studies suggest that referential choices are influenced by animacy. On the one hand, animate referents are more likely to be mentioned as subjects than inanimate referents. On the other hand, animate referents are more frequently pronominalized than inanimate referents. These effects have been analyzed as effects of conceptual accessibility. In this paper, we raise the question whether these effects are driven only by lexical concepts, such that referents described by animate lexical items (e.g., “toddler”) are more accessible than referents described by inanimate lexical items (e.g., “shoe”), or can also be influenced by context-derived conceptualizations, such that referents that are perceived as animate in a particular context are more accessible than referents that are not. In two animation-retelling experiments, conducted in Dutch, we investigated the influence of lexical and perceptual animacy on the choice of referent and the choice of referring expression. If the effects of animacy are context-dependent, entities that are perceived as animate should yield more subject references and more pronouns than entities that are perceived as inanimate, irrespective of their lexical animacy. If the effects are tied to lexical concepts, entities described with animate lexical items should be mentioned as the subject and pronominalized more frequently than entities described with inanimate lexical items, irrespective of their perceptual animacy. The results show that while only lexical animacy appears to affect the choice of subject referent, perceptual animacy may overrule lexical animacy in the choice of referring expression. These findings suggest that referential choices can be influenced by conceptualizations based on the perceptual context.

## Introduction

Throughout the languages of the world, the influence of animacy turns up in numerous linguistic choices. For example, animate entities are more likely to be chosen as the subject or the topic of a sentence than inanimate entities (e.g., Givón, 1983; Dahl and Fraurud, 1996), and they also typically occur earlier in the sentence (e.g., Branigan and Feleki, 1999). The tendency to place animate entities early in the sentence also leads to animacy effects in the choice between alternating grammatical structures. For example, passive sentences are more frequent when the patient role is taken up by an animate entity (McDonald et al., 1993; Van Nice and Dietrich, 2003a). This is illustrated by the preference in English for the sentence in (1a) over the one in (1b) (Prat-Sala and Branigan, 2000).

**Table:** 

(1a)	The woman was run over by the train.
(1b)	The train ran over the woman.

In addition, there is evidence that animacy affects the choice of referring expressions: animate entities have been found to be more often referred to with pronouns than inanimate entities (Dahl and Fraurud, 1996; Yamamoto, 1999; Fukumura and van Gompel, 2011). For example, Fukumura and van Gompel (2011) found in a story completion experiment that speakers were more likely to pronominalize the animate entity (“the hikers”) than the inanimate entity (“the canoes”) in (2a). The same held when grammatical roles were reversed, such as in (2b), suggesting that the effect of animacy on pronominalization is independent of grammatical function. They also found that animacy affected the choice of referent: participants were more likely to refer to the animate NP than to the inanimate NP in their continuations.

**Table:** 

(2a)	The hikers carried the canoes downstream. Sometimes…
(2b)	The canoes carried the hikers downstream. Sometimes…

Thus, animacy appears to influence referential choices: on the one hand, it affects which referent is chosen as the subject of the sentence or as the first-mentioned entity. On the other hand, it affects the type of referring expression that is used to refer to an entity, e.g., a pronoun (“she”) or a full noun phrase (“the girl”). These effects are generally explained as effects of conceptual accessibility (Bock and Warren, 1985): mental representations of animate entities are more easily retrieved from memory than representations of inanimate entities. Therefore, they are available early for linguistic processing (e.g., Prat-Sala and Branigan, 2000), and need less linguistic encoding (Fukumura and van Gompel, 2011). However, it is less clear what the source of these effects is. It could be the case that they arise from the accessibility of lexical concepts (e.g., Branigan et al., 2008), such that the representations associated with animate nouns in the mental lexicon (e.g., “toddler”) are more accessible than those associated with inanimate nouns (e.g., “shoe”). Alternatively, the effects of animacy could be driven by the accessibility of non-linguistic conceptual representations, which may be influenced by the (perceptual) context (e.g., Bock and Warren, 1985; Arnold, 2010). For example, entities that move in a (seemingly) meaningful way may be conceptualized as more animate, and therefore be more accessible. In this paper, we investigate the interplay between a referent’s animacy based on the associated lexical concept (lexical animacy) and the degree to which it is conceptualized as animate or inanimate based on motion cues (perceptual animacy) in referential choices. We investigate effects on both referent choice (which entity is referred to as the subject), and choice of referring expression (whether the entity is referred to with a pronoun or a full noun phrase) in Dutch.

## Theoretical Background

Although strictly speaking the term “animacy” refers to the degree to which something is alive, animacy is not a property of entities in the world. Rather, it is a property of people’s *cognitive representations* of entities, which result from the way people mentally classify entities in the world as “animate” or “inanimate” (a cognitive ontology, Fraurud, 1996). Therefore, this classification differs from a strictly biological sense of “livingness.” For example, in the animacy hierarchy given in (3) (e.g., Comrie, 1989), humans are treated as more animate than animals, although they are not more “alive” in a biological sense.

**Table:** 

(3)	Animacy hierarchy
	Human > Animate > Inanimate

In addition, the way animacy has been found to affect linguistic structure shows that animacy can be a more gradient factor than suggested by the hierarchy in (3). For example, entities such as machines and vehicles, or collectives such as companies and organizations, are treated linguistically as more animate than objects like books and tables (e.g., Comrie, 1989; Dabrowska, 1998; Rosenbach, 2008). Thus, what counts as more animate or inanimate is not so much dependent on properties intrinsic to entities, but on how we *conceptualize* these entities.

In early transformational grammar (e.g., Katz, 1972), animacy was formalized as a semantic feature tied to an entity’s lexical item. A feature that did not match the selection restrictions evoked by the predicate would result in an anomaly. Hence, the sentence in (4) would be anomalous, since “chase” takes an animate subject, while “tree” does not have the feature “animate.”

**Table:** 

(4)	*The tree chased the fly.

Although the anomaly might be resolved in certain contexts, the structure in itself remains ungrammatical under this account. Thus, animacy is regarded here as closely tied to the lexicon. More recently, many (psycho)linguistic studies on animacy also treat the conceptualization of entities only implicitly, presupposing an animate representation for an animate lexical item (e.g., “toddler”), and an inanimate representation for an inanimate lexical item (e.g., “shoe”). For example, Prat-Sala and Branigan (2000) assume that an entity’s animacy contributes to its inherent salience, which is constant across contexts. In addition, Branigan et al. (2008), while considering animacy as one of the factors affecting a referent’s conceptual accessibility, assume that this refers to the accessibility of *lexical concepts*, i.e., concepts that are closely connected to a lexical item.

However, it is clear that people do not always assign the same degree of animacy to the same (lexical) concepts. Like discourse salience (e.g., topichood), this is something that can vary with context. Notably, in some contexts people can conceptualize usually inanimate entities as animate. In cartoons or fairy tales, for example, inanimate entities or animals are often anthropomorphized. This also happens in real-world contexts, as when someone says “The tree wants to catch me” for a tree with branches sticking out like arms. The reverse, treating animate entities as inanimate, is theoretically also possible, although this may be less likely^1^. In addition, in figurative language use such as personification, metaphor, and metonymy, entities are often referred to in a way that does not match their actual animacy, as in “His ideas will live on forever” (Lakoff and Johnson, 1980), or “The ham sandwich is sitting at table 20” (Nunberg, 1979), to refer to a customer in a restaurant.

Evidence that there is variation in the way entities are conceptualized as animate or inanimate comes from different areas of research. From linguistic typology we know that languages differ in which entities are treated as animate or inanimate in the grammar. A well-known example is that of the Algonquian language Fox, in which the word for “strawberry” is grammatically inanimate, while the word for “raspberry” is animate (Anderson, 1997). Hence, the former cannot occur as the beneficiary role in ditransitive constructions, while the latter can. Similarly, in Persian, the word for “tree” is lexically classified as animate, by which it takes the animate plural suffix, while the word for “flower” takes an inanimate suffix (Wiese, 2003). In addition, in many European languages inanimate nouns have masculine or feminine gender, which may affect how they are conceptualized (e.g., Dahl, 2000; Boroditsky et al., 2003). There is also evidence that conceptualizations of animacy may differ across contexts within the same language. In an ERP-study by Nieuwland and Van Berkum (2006), utterances that would normally violate animacy requirements, such as “The peanut was in love,” were found to be easy to process when they were embedded in a fairy tale or cartoon-like context, in which inanimate objects were consistently made the subject of predicates that require an animate subject (e.g., *dance*). Within such contexts, a normally well-formed utterance such as “The peanut was salted” became more difficult to process. This suggests that people can easily accommodate to contexts in which normally inanimate objects are presented as animate, and that these objects are treated as fully animate from that point on. This makes it clear that the classification of concepts according to their lexical-semantic animacy should be distinguished from contextually inferred animacy (cf. Yamamoto, 1999; Rosenbach, 2008).

The conceptualization of entities as more or less animate in reference may be related to the anthropocentric nature of language, i.e., people talk about things from their own, human, perspective. An entity may thus be more animate the more it resembles humans. The reason for this may be that people have more empathy toward such entities (e.g., Kuno and Kaburaki, 1977), or find them otherwise more important to talk about (Givón, 1983). Sridhar (1988), for example, found that when people described an interaction between a ball and a doll (two inanimate objects), the doll was more likely to be mentioned first in the sentence. It has been suggested that the relevant property in conceptualizing referents is degree of individuation, e.g., whether they appear to be autonomous beings, have the ability to act upon their environment, or have goals, intentions, and mental states (Fraurud, 1996; Dahl, 2008). An important factor in classifying an entity as an individual is how it is perceived. For example, individuals typically exist in their own right (they are not physically part of another entity), they move without the intervention of an external force, and they act in meaningful ways. Hence, a clear perceptual cue for animacy or individuation is motion. According to the perception literature, movements of simple geometric objects can indeed induce a strong and immediate percept of animacy (e.g., Scholl and Tremoulet, 2000). In an early study by Heider and Simmel (1944), participants were found to readily assign emotions and intentions to geometric objects when they moved in non-random ways. More recent work shows that even very subtle movement cues can still create a perception of animacy. For example, a sudden change in speed or direction already leads to animate percepts (Tremoulet and Feldman, 2000). In addition, when the movements of two objects are correlated or one moving object pauses near another object, this creates a suggestion of animacy (Schultz et al., 2005; Santos et al., 2008).

Applied to normally inanimate entities, such perceptual motion cues may cause them to be conceived of as more animate and more individuated, which may make them more conceptually accessible. According to the theory of conceptual accessibility (Bock and Warren, 1985), the activation of mental representations of referents in memory is fed by both perception and conceptual knowledge. Indeed, Bock et al. (1992) and McDonald et al. (1993) found that animacy effects on word order were enhanced when participants created a mental image of the entities. Similarly, in the Nieuwland and Van Berkum (2006) study most participants reported to have visualized the story and to have seen the inanimate objects as cartoon-like characters with human characteristics such as a face, arms, and legs. This suggests that context-dependent perceptual information, such as motion, can contribute to a referent’s conceptual accessibility, on top of conceptual information from long-term memory. Prat-Sala and Branigan (2000) distinguish two types of accessibility: inherent accessibility, which concerns properties of a referent that remain stable across contexts, and derived accessibility, which concerns the salience of a referent in the linguistic or non-linguistic context. Factors influencing inherent accessibility typically include lexical animacy (e.g., Prat-Sala and Branigan, 2000) and concreteness (Maes, 1997), while derived accessibility is typically affected by factors such as givenness (e.g., Ferreira and Yoshita, 2003) and thematic role (Van Nice and Dietrich, 2003b). Since perceptual motion cues for animacy may change across contexts, and are not intrinsic to the entity itself, we may add perceptual animacy as a factor contributing to derived accessibility.

In Prat-Sala and Branigan’s (2000) view, a referent’s overall conceptual accessibility is a combination of its inherent and its derived accessibility. They also argued, on the basis of a picture description experiment, that in strong enough contexts, derived accessibility might override effects of inherent accessibility. Hence, a lexically animate entity is more likely to be mentioned in subject position than an inanimate entity, unless the discourse makes the inanimate entity salient enough to overcome the difference in inherent accessibility. Additional evidence that derived accessibility may override inherent accessibility has been found, e.g., by Christianson and Ferreira (2005) and Van Nice and Dietrich (2003b). This is also consistent with the findings of Nieuwland and Van Berkum (2006), who found that the lexical meaning of “peanut” was overruled by the pragmatic inference of the referent’s animacy due to the discourse context. On the other hand, other studies have not found evidence for the dominance of one type of accessibility over the other (e.g., Van Nice and Dietrich, 2003b; Fukumura and van Gompel, 2011).

The question we ask in this paper is how lexical-semantic animacy and contextually driven animacy interact in determining referential choices in language production. To address this question, the present study investigates the interplay between the animacy associated with lexical concepts (henceforth *lexical animacy*), and the perceived animacy based on the referent’s movements (henceforth *perceptual animacy*) in Dutch spoken language production. That is, we investigate whether lexically inanimate referents that are conceptualized as animate and lexically animate referents that are conceptualized as inanimate are different from the congruent cases with respect to referential choices. We examine both the choice of referent (which entity is referred to as the subject) and the choice of referring expression (use of pronouns and full noun phrases).

One possible hypothesis is that lexical and perceptual cues for animacy both affect the conceptual accessibility of a referent, but that perceptual animacy overrules lexical animacy, in line with Nieuwland and Van Berkum (2006). Hence, animate moving objects should be more likely to be referred to as subjects and with pronouns than inanimate moving objects, irrespective of lexical animacy. Only when the entity is perceptually inanimate, lexical animacy is expected to have an effect, since conceptualizing animate entities as inanimate may be less straightforward.

Alternatively, lexical and perceptual animacy may affect accessibility independently. In this case, a referent’s accessibility is predicted to be highest when the entity is both mentioned using an animate lexical description and perceived as animate. It should be lowest when the referent is both lexically and perceptually inanimate. In the incongruent cases, i.e., lexically animate but perceptually inanimate or vice versa, accessibility is predicted to be intermediate. Assuming that both the rate of pronominalization and the likelihood of being mentioned as the subject increase proportionally with an increase in accessibility, pronouns, and subject references are predicted to be most frequent in cases where all cues point to a high degree of animacy, to be least frequent in cases where all cues point to a low degree of animacy, and somewhere in between for the incongruent cases. If conceptualizing lexically inanimate objects as animate is easier than conceptualizing lexically animate objects as inanimate, the effect of perceptual animacy should be at least present in the lexically inanimate condition.

We conducted two experiments, in which participants watched animations of simple geometric objects, such as circles and triangles, and retold them afterward. We used retelling from memory rather than speaking when the animations were still in view because we believed this would be the more natural communicative situation (cf. Christianson and Ferreira, 2005). The perceptual animacy of the objects in the animations was manipulated by using movement cues to create animate and inanimate conceptualizations. Manipulating motion allowed us to make use of the exact same objects in the animate and in the inanimate conceptualizations. In this way, the appearance of the referent was kept constant across all conditions, such that it could not influence the referent’s perceptual or lexical animacy. In addition, we separated lexical animacy from perceptual animacy by giving lexical labels to the objects. In Experiment 1, the lexical labels were animate and inanimate nouns that either matched or did not match in animacy with the movements. In Experiment 2, we replaced the lexical labels with nonsense words that could be interpreted as either referring to animate or referring to inanimate entities, to exclude a possible influence of lexical animacy on perceptual animacy.

## Experiment 1

### Materials and methods

#### Participants

Sixty-four students from Tilburg University participated in this experiment for course credit. All were native speakers of Dutch. All participants gave their consent to the use of their data.

#### Materials

**Figure 1:**
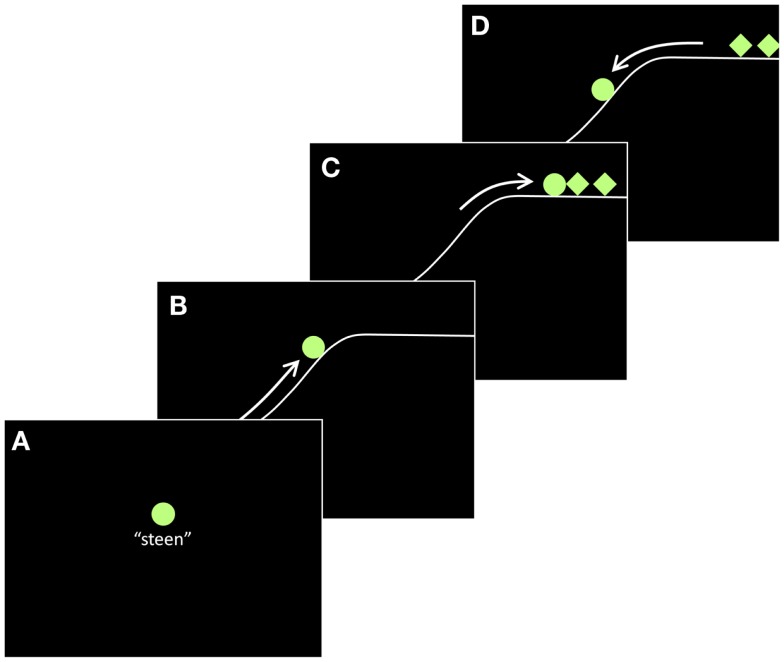
**Example of an incongruent stimulus item in Experiment 1, with the target figure moving in an animate manner, but having the inanimate lexical label *steen* “stone.”** The four frames **(A–D)** are stills taken from a continuous animation. Letters indicate order; arrows indicate movement. Both were not shown in the experiment.

We created 16 different animations, using the motion paths from the custom animation function in Microsoft PowerPoint. Each animation featured three geometric objects, of which one was the target figure and the two others were competitors. The objects were selected from the following built-in shapes in Powerpoint: cross, oval, rectangle, isosceles triangle, up arrow, and diamond. All had the same dimensions. The two competitor objects both had the same shape, which was always different from the target figure’s shape. The figures appeared in one of four colors: white, light green, light blue, or light purple. Within one animation, colors of the target and the competitors were always the same. Shapes and colors were assigned randomly to the animations, except for animations involving rolling or bouncing movements, in which the target object was always a circle. Eight animations contained animate motion of the target figure, and the other eight contained inanimate motion of the target figure (to be explained below). The animate and inanimate animations were paired, such that for each animate animation there was another animation featuring the same objects but in which the target figure moved in an inanimate way. An example of an animate stimulus item is given in Figure 1. The animations were presented on a black background, but some animations included the suggestion of a landscape, presented by a white continuous line (as in Figure 1). This was done to aid the interpretation of some movements (e.g., “climbing up a slope” instead of “taking off magically into nothingness”).

Each target figure was given a linguistic label (in Dutch), either animate (e.g., *padvinder* “boy scout”) or inanimate (e.g., *steen* “stone”). The animate and inanimate labels were matched (across items) for frequency and number of characters. The complete list of lexical labels can be found in Table A1 in the Appendix. The competitors had no labels. In each trial, the target figure was presented just before the start of the animation along with its label (Figure 1A). To make repeated references possible, each animation consisted of three “episodes.” First, the target figure performed an intransitive action (Figure 1B). Here, the target figure moved either in an animate way or in an inanimate way. Animate movements were suggested by simulating self-propelled actions (e.g., climbing up a slope), using cues such as changes in speed or direction (Tremoulet and Feldman, 2000). The animate movements used were: moving back and forth horizontally across the screen at varying speeds (two animations); jumping up and down irregularly; hopping back and forth across the screen with varying intervals (two animations); moving up a slope with a pause just before the top; moving diagonally up across the screen in small steps; making irregular arc-shaped movements across the screen. Inanimate movements were suggested by creating the impression that they were caused by an external (invisible) force such as gravity (e.g., rolling down a slope; Gelman et al., 1995). Since we wanted to keep implicit what set the object in motion, inanimate movements necessarily started off-screen. The inanimate movements used were: moving across the screen at a constant speed (i.e., as if sliding on ice; two animations); bouncing vertically several times with loss of energy; bouncing once (two animations); rolling down a slope; whirling from top to bottom; moving down and up the slopes of a valley with loss of energy.

Second, the competitor figures entered the screen, and the target figure performed a transitive action [interaction with competitor figures (Figure 1C)]. Animate movements included: colliding with the competitors from rest; jumping on and off the competitors (two animations); pushing the competitors away (two animations); quickly jumping up and down in front of the competitors (as if startled); bumping into the competitors; briefly touching the competitors. Inanimate movements included: colliding with the competitors and bouncing back (two animations); land on top of the competitors (three animations); colliding with the competitors and pushing them away (two animations); bouncing over the competitors. The competitors were included to allow alternating syntactic structures (e.g., “the stone hits the two hikers” vs. “the two hikers are hit by the stone”), as well as to encourage reference switches, which should lead to variation in referring expression use (both pronouns and full noun phrases). They appeared in dyads, such that pronominal references to the target or the competitors were likely to be unambiguous (singular vs. plural). The only movement that the competitors made was sliding into the screen (either from the left or the right). Since this movement was not particularly animate or inanimate, the perceptual animacy of the competitors remained ambiguous. The target figure always appeared before the competitors, to make it a likely candidate for the discourse topic. To control for agency, the target figure was always the agent in the transitive action, both in the animate and in the inanimate conditions.

Finally, the target figure performed another intransitive action (Figure 1D). Animate movements included: (quickly) moving off the screen from rest (four animations); hopping off the screen from rest; rolling down the slope, off the screen; quickly hopping or stepping down diagonally, off the screen (two animations). Inanimate movements included: moving off the screen; being bounced off the screen; landing on the ground while turning on its axis (three animations); bouncing back and coming to a rest (three animations).

The animations (without the lexical labels) were pretested for perceived animacy of the target referent in a perception study. Eight participants were asked to rate the target referent in each of the animations for animacy on a seven-point Likert scale, with one being “clearly lifeless” and seven being “clearly alive” (cf. Tremoulet and Feldman, 2000). Ratings were given for each animation as a whole, not for each movement separately. The results confirmed that animations intended to be animate were scored significantly higher (*M*_anim_ = 5.13; *M*_inan_ = 2.53; One-way ANOVA: *F*(1,126) = 114.48, *p* < 0.001, MSE = 1.88).

#### Procedure

The participants were seated at a table, facing the experiment leader, who sat in a chair facing the participant. The experiment was presented on a laptop, which was on the table at an angle with the participant. The participants’ task was to retell the animations to the experiment leader. Before each animation, the target figure was presented in the middle of the screen, accompanied by its lexical label. The target figure and the label were then replaced by a crosshair, after which the animation started. Participants watched each animation twice, so that they could accurately retell them from memory. They were not allowed to start talking when the animation was still running, because this may have caused them to skip over crucial information. The participants were instructed to use the label presented in the beginning when mentioning the target figure. (Of course, participants were allowed to pronominalize referents. Although it was not instructed explicitly, all participants did this.) The competitors could be referred to in any way they wanted. To ensure lively retellings, participants were further instructed to retell the animations “in a fanciful manner, as if telling it to a child.” The experiment started with three practice trials, after which any remaining questions could be asked. The experiment leader gave only minimal feedback during the experiment (e.g., nodding or saying “Okay” after each trial; in a few cases the participant received some encouragement to start talking). There were no further interactions between participant and experiment leader while the experiment was running. It took about 25 min to complete the experiment.

#### Design

Crossing the factors lexical animacy and perceptual animacy resulted in a 2 (lexically animate, lexically inanimate) × 2 (perceptually animate, perceptually inanimate) within-participants and within-items design. Participants were randomly assigned to one of four lists, which were created such that from a given item each condition occurred on a different list. The items were presented in a random order. The same order was used across all lists.

#### Data coding and statistical analyses

The data from one participant were discarded, because this person retold the animations while watching them instead of afterward. The data from the other participants were coded by the first author. Uncertainties were resolved through discussion with the other authors. First, all stories were divided into fragments containing descriptions of the three episodes (initial intransitive action, transitive action, and final intransitive action). We focused on the descriptions of the transitive action, since these were the fragments that were expected to show most variation in choice of referent for the subject position and choice of referring expression. Next, the fragments were coded for whether the target figure was made the subject of the critical clause (referent choice), and whether the target figure was referred to using attenuated expressions (choice of referring expression). We coded all grammatical subjects of both main and subordinate clauses as “subject,” and everything else was coded as “object.” We defined attenuated expressions as all referring expressions that were not full noun phrases. These included full pronouns (e.g., *zij* “she”), reduced pronouns (e.g., *ze* “she”), demonstrative pronouns (e.g., *die* “that one”), and zero anaphora (e.g., … *en Ø springt over twee huizen* “… and Ø jumps over two houses”). Henceforth, we will use the term “pronoun” to refer to all these types of referring expressions. If there was more than one clause describing the action, we only coded the first one. Trials in which the transitive action was not described were excluded from analysis. In addition, we excluded trials in which reference was made to a person or object that was not present in the animation, since this could have altered the interpretation of the referent’s animacy, as well as its discourse accessibility. Finally, we excluded trials in which the target figure was referred to with an indefinite NP (generally used in contexts where a pronoun is not possible), referred to with a proper noun (typically used for animate referents), or not referred to at all. In all, 96 cases (19.0%) were removed. We controlled for discourse salience by coding whether the target figure was mentioned in the sentence directly preceding the clause under consideration, and if so, in what grammatical function.

We analyzed the data using logit mixed models (Jaeger, 2008). Lexical animacy and perceptual animacy were included as fixed factors; participants and items were included as random factors. Fixed factors were centered to reduce collinearity. Two analyses were carried out: one on the log odds of a subject reference, and one on the log odds of a pronoun reference. Starting with the full random effect specification, we omitted random slopes that did not significantly affect the model’s fit using model comparison. We present only the final models.

### Results

#### Choice of referent

**Figure 2:**
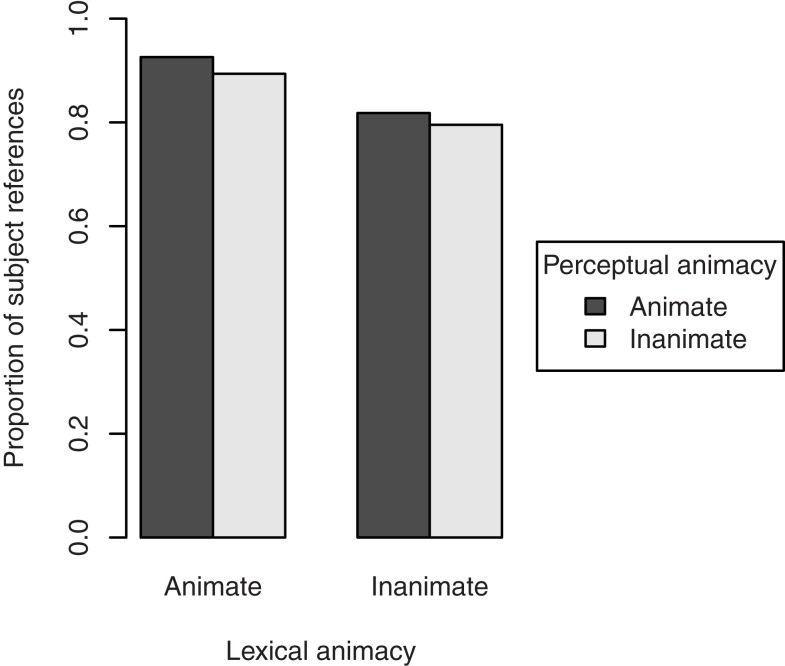
**Proportion of subject references to the target figure in the description of the transitive action in Experiment 1, by its lexical and perceptual animacy**.

Figure 2 shows the proportion of references to the target figure as the subject of the clause describing the transitive action. Lexically animate target figures were mentioned as the subject in 91.0% of the cases, whereas lexically inanimate target figures were mentioned as the subject in 80.7% of the cases. The effect of lexical animacy on choice of referent was significant, β = 0.93, SE = 0.31, *p* < 0.01. We found no effect of perceptual animacy, β = 0.29, SE = 0.32, *p* = 0.37, and no interaction, β = 0.28, SE = 0.62, *p* = 0.65. Random slopes were not included, since they did not improve the model’s fit^2^.

**Figure 3:**
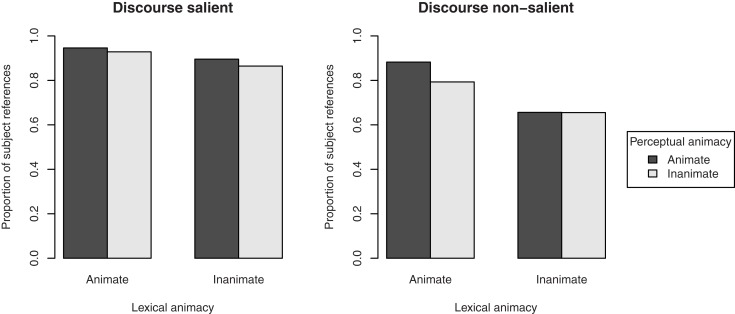
**Proportion of subject references to the target figure in the description of the transitive action in Experiment 1, by its lexical and perceptual animacy, and split by the discourse salience of the referent: mentioned in the directly preceding sentence (discourse salient, left pane), or not mentioned in the directly preceding sentence (discourse non-salient, right pane)**.

To examine whether the effect of lexical animacy was confounded by the salience of the target figure in the discourse, we performed separate analyses for the cases in which the referent was mentioned in the directly preceding sentence (*n* = 284), and for the cases in which the referent was not mentioned in the directly preceding sentence (*n* = 124)^3^. The results, presented in Figure 3, show that the effect of lexical animacy only holds when the referent was not mentioned in the previous sentence (discourse non-salient), β = 1.07, SE = 0.45, *p* < 0.05. The difference between lexically animate and inanimate referents that were discourse salient was not significant, β = 0.74, SE = 0.45, *p* = 0.10. Again, perceptual animacy was non-significant in both data sets, β = 0.42, SE = 0.47, *p* = 0.37 and β = 0.30, SE = 0.46, *p* = 0.52, respectively; interactions: β = 0.70, SE = 0.91, *p* = 0.44 and β = −0.03, SE = 0.91, *p* = 0.97, respectively.

#### Choice of referring expression

**Figure 4:**
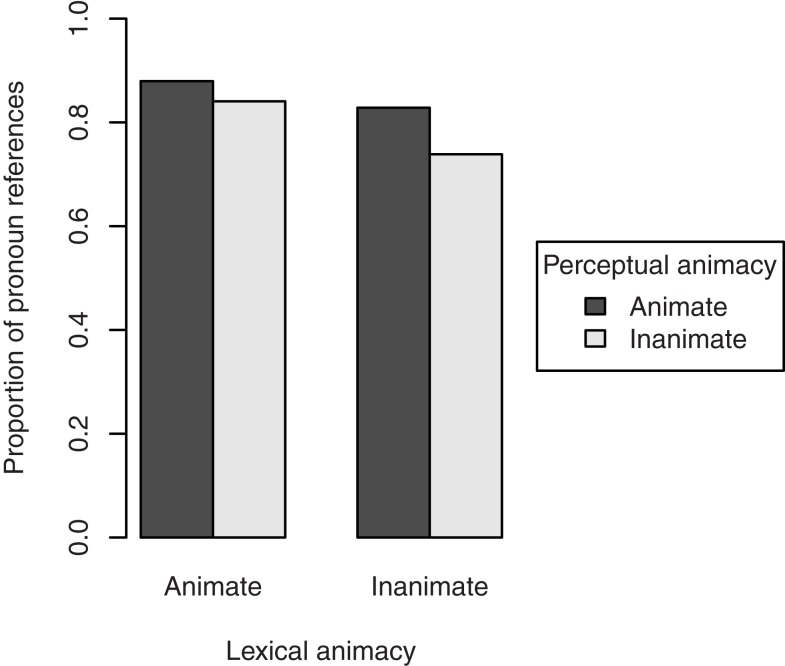
**Proportion of pronoun references to the target in the description of the transitive action in Experiment 1, by its lexical and perceptual animacy**.

Figure 4 shows the overall proportion of pronoun (i.e., non-full NP) references to the target figure in the description of the transitive action. This includes both subject and object pronouns. Lexically animate referents were referred to with pronouns in 86.0% of the cases, against 78.6% for lexically inanimate referents. The effect of lexical animacy was significant, β = 0.58, SE = 0.28, *p* < 0.05. In addition, perceptually animate referents were referred to with pronouns in 85.5% of the cases, against 79.6% for perceptually inanimate referents. Although this effect was just slightly smaller than that of lexical animacy, it was only marginally significant, β = 0.48, SE = 0.28, *p* = 0.09. We found no interaction between lexical and perceptual animacy, β = −0.22, SE = 0.55, *p* = 0.69. Random slopes were not included, since they did not contribute to the model’s fit^4^.

**Figure 5:**
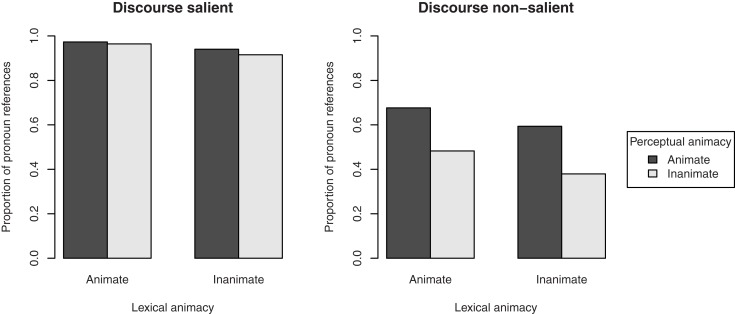
**Proportion of pronoun references to the target figure in the description of the transitive action in Experiment 1, by its lexical and perceptual animacy, and split by the discourse salience of the referent: mentioned in the directly preceding sentence (discourse salient, left pane), or not mentioned in the directly preceding sentence (discourse non-salient, right pane)**.

To investigate whether these effects were confounded with the referent’s discourse salience, we performed two separate analyses, one for the cases in which the referent was mentioned in the directly preceding sentence (*n* = 284), and one for the cases in which it was not mentioned (*n* = 124). The results, presented in Figure 5, show that when the referent was discourse salient (i.e., mentioned in the previous sentence), no effects of animacy were present (lexical animacy: β = 0.90, SE = 0.60, *p* = 0.14; perceptual animacy: β = 0.31, SE = 0.62, *p* = 0.62; interaction: β = −0.05, SE = 1.20, *p* = 0.97). However, when the referent was discourse non-salient (i.e., not mentioned in the previous sentence), perceptual animacy had a significant effect on the choice of referring expression, β = 1.35, SE = 0.47, *p* < 0.01: more pronouns were used when the referent was perceptually animate. The effect of lexical animacy was no longer significant, β = 0.66, SE = 0.46, *p* = 0.15, suggesting that this factor may indeed be partly confounded with discourse salience (i.e., what is lexically animate is also more likely to be the subject, cf. Figure 2). There was no interaction, β = −0.58, SE = 0.91, *p* = 0.52.

**Figure 6:**
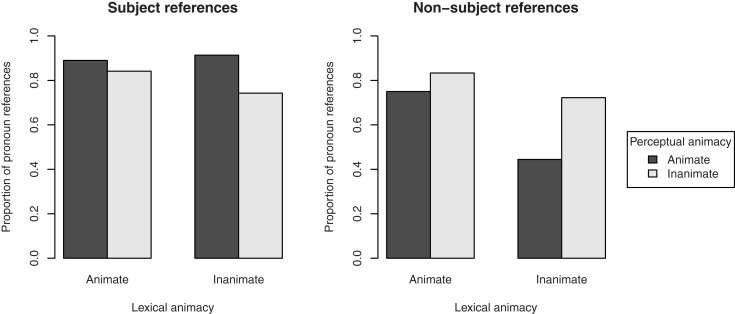
**Proportion of pronoun references to the target figure in the description of the transitive action in Experiment 1, by its lexical and perceptual animacy, and split by the grammatical function of the referent: subject (left pane), or non-subject (right pane)**.

We also investigated whether the grammatical function of the referent in the current sentence showed the same confound. To this end, we performed separate analyses on referring expressions in subject position (*n* = 352) and non-subject position (*n* = 56). The results, presented in Figure 6, showed a significant effect of perceptual animacy on the choice of referring expression in subject position, β = 0.89, SE = 0.34, *p* < 0.01: more pronouns were used when the referent was perceptually animate. There was no effect of lexical animacy, β = 0.22, SE = 0.34, *p* = 0.51, and no interaction, β = −0.91, SE = 0.68, *p* = 0.18. Although Figure 6 suggests an effect of perceptual animacy in the opposite direction in non-subject position, this was not significant, β = −0.82, SE = 0.69, *p* = 0.24. The same held for lexical animacy, β = 0.99, SE = 0.67, *p* = 0.14. There was no interaction, β = 0.67, SE = 1.33, *p* = 0.62. Again, these patterns suggest a confound of discourse salience/grammatical function with lexical animacy, but not with perceptual animacy.

### Discussion

The results of Experiment 1 showed differential effects of the two types of animacy manipulations. Firstly, in descriptions of the transitive event in the animations, which involved an interaction between the target figure and the two competitor figures, the target figure’s lexical animacy, but not its perceptual animacy, influenced whether it was mentioned as the subject of the sentence or not. For example, participants were more likely to say *de padvinder duwt de pestkoppen weg* “the boy scout pushes the bullies away” than they were to say *de steen duwt de pestkoppen weg* “the stone pushes the bullies away” (in which case sentences such as *de pestkoppen worden weggeduwd door de steen* “the bullies are pushed away by the stone” were more frequent)^5^. However, whether it was an animate-like moving stone (e.g., one trying to climb up a slope) or not did not matter. In addition, the effects of animacy were only present when the referent was not mentioned in the previous sentence, suggesting that discourse salience is a stronger factor in determining which grammatical role is assigned to the referent.

Secondly, the choice of referring expression to refer to the target figure in the transitive action seemed to be affected by perceptual animacy: more pronouns were used when referents were moving in an animate way, independently of their lexical animacy. For example, participants were more likely to say *hij duwt ze weg* “it pushes them away” than they were to say *de steen duwt ze weg* “the stone pushes them away,” when the referent was an animate moving stone. Again, this effect was confined to the cases in which the referent was discourse non-salient; when the referent was discourse salient, participants used pronouns almost exclusively, masking any effects of animacy. In addition, the effect of perceptual animacy was also confined to the cases in which the referent was the subject of the current sentence, suggesting that the environments in which perceptual animacy becomes relevant are cases of topic shift. Although pronominalization of lexically animate referents was also somewhat more frequent than pronominalization of lexically inanimate referents, this difference was not significant when controlling for discourse salience, suggesting that the effect of lexical animacy may be partly indirect, i.e., lexically animate referents are more likely to be subjects, and this in turn increases pronoun use. These findings show that the choice of referring expression can be influenced by factors induced by the non-linguistic context, such as perceptual animacy. Surprisingly, the effect of perceptual animacy was equally large in the lexically animate and lexically inanimate condition, suggesting that lexically animate referents can as easily be conceptualized as inanimate as lexically inanimate referents can be conceptualized as animate.

In Experiment 1, we tried to manipulate perceptual and lexical animacy independently. The results suggest that the two factors affect choice of referent and choice of referring expression differently. In the choice of referring expression, perceptual animacy seems to be a stronger cue for accessibility than lexical animacy, at least when discourse salience is low. Although lexical animacy does not seem to be completely overruled, this finding is in line with Nieuwland and van Berkum’s (2006) findings that in case of a conflict between the two factors, perceptual animacy gets prevalence. Thus, a stone that is trying to climb up a slope is assigned animacy because of its animate-like movements. In the choice of referent for the subject position, however, only lexical animacy seems to have an effect.

It is possible, however, that the lexical items influenced perception in the experiment, and that therefore the two factors were not independent. For example, while an animation of a circle bouncing in a very regular manner is likely to be interpreted as inanimate movement (a bouncing ball), calling the circle a “prince” may encourage the viewer to find an interpretation of the movement that matches the animacy of the lexical item. In this example, one could come up with a story about a prince jumping on a trampoline (and some participants did). Similarly, a circle trying to climb up a slope might look very animate-like, but calling the circle a “stone” may cause the viewer to come up with an interpretation in which the stone was pushed with such force that it could withstand gravity and roll upward. This makes it unclear whether the factor perceptual animacy really measured what it should measure, namely the impression of animacy people would get from purely perceptual features, i.e., movements. Experiment 2 was set up to deal with this potential complication.

## Experiment 2

Experiment 2 was similar to Experiment 1, except that we replaced the lexical labels by nonsense words. These words were chosen in such a way to avoid intuitions about the animacy of the word as much as possible. In this way, we expected to minimize the chance that the lexical labels would influence the interpretation of the movements, and to get a clearer picture of the effects of perceptual animacy on referential choices.

### Materials and methods

#### Participants

Fifteen undergraduate students from Tilburg University participated in this study as speakers. Another 15 naive participants acted as addressees. Ten speakers and nine addressees participated for course credit; the others volunteered. All gave their consent to the use of their data. None of them had participated in Experiment 1.

#### Materials

The animations were identical to those used in Experiment 1. However, instead of real words, the lexical labels consisted of nonsense words. These words were constructed by altering the real words from Experiment 1, while keeping the length in characters and the number of syllables the same (e.g., *daptinder* from *padvinder* (“boy scout”). Together with a number of real words, these constructed words were entered into a pretest in which nine participants indicated for each word whether they knew the word or not, and if not, to what degree they thought the word could refer to a person, an animal, or a thing. Participants marked their answers on five-point Likert scales (e.g., “very likely a person” to “very likely NOT a person”). Eight nonsense words that were indicated as “unknown” by all participants, and had average scores around the middle of all three scales were selected for the present experiment. As in Experiment 1, they were presented together with the target figure just before the start of each animation. To ensure that the labels would be interpreted as nouns referring to the target figures, the labels were preceded by the phrase *dit is een* “this is a.” A list of all nonsense words can be found in Table A2 in the Appendix.

#### Procedure

The procedure was similar to that of Experiment 1, except that the addressee for the story retellings was no longer the experiment leader, but another naive participant. This was done to make the stories even more lively, which should reduce the number of missing data. To make the task more engaging, we also gave the addressees a task. For each object described by a nonsense word, they had to indicate on an answer sheet whether they thought this object was a person, an animal, or a thing. Because instructions were only given in written form, the speakers remained unaware of the nature of this task. Four speaker-addressee couples were tested in a face-to-face setting similar to that in Experiment 1; the other 11 couples communicated through Eye Catchers^6^, because they were tested directly after another, unrelated, experiment that used this setup. Instructions were virtually identical to those of Experiment 1. After two practice trials, the experiment was started and the experiment leader left the room. It took about 20 min to complete the experiment.

#### Design

Since lexical animacy was held constant in this experiment by using nonsense words, the only independent variable was perceptual animacy. Participants were randomly assigned to one of two lists, which were created such that from a given item the perceptually animate version occurred on one list, and the perceptually inanimate version on the other. Items were presented in a random order. The same order was used in the two lists.

#### Data coding and statistical analyses

The coding scheme and the procedure for statistical analysis were identical to that of Experiment 1. We excluded six cases (5.0%) because either the transitive action was not described, or the given lexical label was not used.

### Results

#### Choice of referent

**Figure 7:**
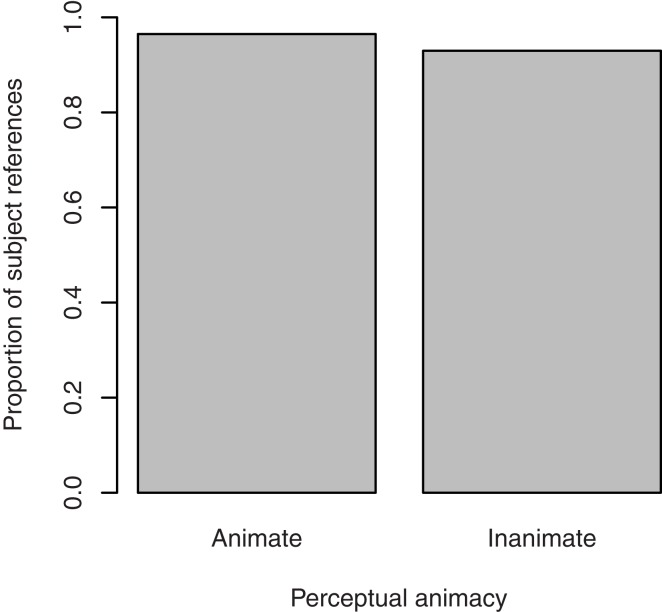
**Proportion of subject references to the target figure in the description of the transitive action in Experiment 2, by its perceptual animacy**.

Figure 7 shows the proportion of references to the target figure as the subject of the clause describing the transitive action as a function of perceptual animacy. Target referents were made the subject of the sentence in the great majority of the cases (perceptually animate referents: 96.5%; perceptually inanimate referents: 93.0%). There was no significant effect of perceptual animacy, β = 0.95, SE = 1.03, *p* = 0.36. Random slopes were not included, since they did not improve the model’s fit^7^.

#### Choice of referring expression

**Figure 8:**
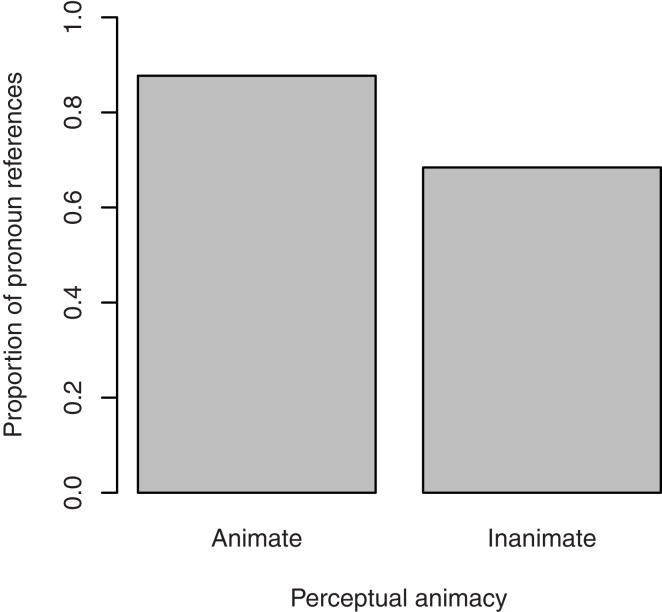
**Proportion of pronoun references to the target figure in the description of the transitive action in Experiment 2, by its perceptual animacy**.

Figure 8 shows the proportion of pronoun references to the target referent in the descriptions of the transitive action as a function of perceptual animacy. Pronouns were used more frequently when the target referent was perceptually animate (87.7%) than when it was perceptually inanimate (68.4%). The effect of perceptual animacy was significant, β = 1.31, SE = 0.52, *p* < 0.05. Random slopes were not included, since they did not improve the model’s fit^8^.

### Discussion

The aim of Experiment 2 was to exclude the possibility that the perceived animacy of referents is influenced by the animacy of their lexical descriptions. By using nonsense words for which people have no strong intuitions about animacy, we controlled for this possible influence. The results largely confirmed the results of Experiment 1. An effect of perceptual animacy was found on the choice of referring expression in the descriptions of the transitive action. Here, perceptually animate referents were more likely to be pronominalized than perceptually inanimate referents. As before, perceptual animacy did not affect the choice of whether the referent was placed in subject position. These findings again suggest that perceptual animacy, a factor that is dependent on the visual context, can influence the choice of referring expression.

## General Discussion

This study was conducted to investigate whether perceptual animacy of referents influences referential choices, and how this interacts with lexical animacy. Firstly, the results of Experiment 1 confirm that lexical animacy affects the choice of referent for the subject position: lexically animate referents were more likely to be mentioned as the subject of a transitive sentence. This is in line with research indicating that animacy affects grammatical function assignment (Bock et al., 1992; McDonald et al., 1993; Dahl and Fraurud, 1996). The effect was confined to contexts in which the referent was not discourse salient, suggesting that discourse salience may overrule effects of animacy (cf. Prat-Sala and Branigan, 2000). Perceptual animacy, on the other hand, did not seem to influence the choice of referent for the subject position, even when lexical cues for animacy were not present (Experiment 2). This suggests that visual cues can be overridden by lexical cues, which is not what would be predicted under the assumption that derived accessibility can override inherent accessibility.

Secondly, lexical animacy influenced the choice of referring expression: also in line with previous research (Dahl and Fraurud, 1996; Yamamoto, 1999; Fukumura and van Gompel, 2011), lexically animate referents were more frequently pronominalized, although this effect might be partly mediated by grammatical function. More importantly, the results of both Experiment 1 and Experiment 2 show that in addition to lexical animacy, perceptual animacy also affects pronominalization. This supports the hypothesis that both cues for animacy affect a referent’s conceptual accessibility. As predicted, perceptual animacy turned out to be a stronger factor in determining the choice of referring expression than lexical animacy, at least for discourse non-salient referents. This suggests that in this case visual cues can override lexical cues, in line with the idea that derived accessibility can override inherent accessibility. This is also consistent with Nieuwland and Van Berkum (2006), who found that contextual cues overruled lexical-semantic cues in comprehension. Surprisingly, in our study this does not only hold for lexically inanimate objects, which can be conceptualized as animate in a certain context, but also for lexically animate entities, which seem to become less animate when they move in an inanimate way.

While our materials were abstract, people readily conceptualized objects as more animate or more inanimate based on motion cues, as was already shown in the animacy pretest. That participants easily accommodated their conceptualizations of objects to the perceptual context, independently of their lexical animacy, can also be seen from the way the objects were described in Experiment 1. For example, lamps and snowflakes were described as “happy” or “afraid,” and a handbag “tries” or “decides” to go in a certain direction. Conversely, there were some indications that lexically animate objects were conceptualized as inanimate. Besides the use of predicates that are associated (but not exclusively) with inanimate movement, such as “fall,” “bounce,” and “slide,” there were also a few cases in which objects were referred to with pronouns of which the gender did not match with the noun (e.g., using *hij* “he,” “it” to refer to a queen). Although these cases might be errors, they were confined to the lexically animate-perceptually inanimate condition, suggesting that conceptual information associated with the lexical item was overruled by a different conceptualization. However, given the gradual nature of animacy (Comrie, 1989), most objects were probably conceptualized as neither fully animate nor fully inanimate, both in the experiment and in the animacy pretest. The crucial point is, however, that manipulating motion alone made some objects appear more animate than others, and the same manipulation also affected pronoun use. Hence, objects that were statistically more likely to be conceptualized as animate were statistically more likely to be pronominalized.

These results have several theoretical implications. First of all, they suggest that which entity becomes the subject of the sentence is affected by the animacy of the lexical items, but not by the conceptualization of the entity based on perceptual cues. This seems inconsistent with a conceptual accessibility account of linearization (e.g., Bock and Warren, 1985; Prat-Sala and Branigan, 2000; Van Nice and Dietrich, 2003a). According to such an account, it is the conceptual (rather than the lexical) accessibility of animate entities that causes them to be mentioned earlier in the sentence (either through a direct link to word order, or indirectly via grammatical function). Consequently, a lexically inanimate entity that is conceptualized as animate, such as a stone climbing up a slope should also be more accessible according to this account. However, others have linked conceptual accessibility to the retrieval of lexical items (Levelt et al., 1999; Branigan et al., 2008). According to Levelt et al. (1999), there is a close connection between concepts, lemmas, and word forms. When a concept is highly accessible, this speeds up lemma retrieval, and, assuming incremental sentence production, the corresponding lexical item is produced first. This might explain the dominance of lexical animacy in referent choice. A crucial factor here might be the nature of the task. In our experiments, the lexical labels were presented before the start of the animations. Therefore, in Experiment 1 the concepts associated with the lexical items were already activated before any conceptualizations on the basis of perceptual cues were made. Hence, any contextually induced conceptualizations could have been overruled by the animacy of the activated lemmas when participants mentioned the referents. Our results might therefore have been different if we had presented the lexical labels after the animations.

In addition, Van Nice and Dietrich (2003b) found that when participants described pictures that remained in view, the animacy of the agent and the animacy of the patient both affected the rate of passivization, but there were no interactions between the two factors, suggesting that speakers were not actively comparing properties of different entities. However, when they had the participants describe the pictures from memory, they did find an interaction. Van Nice and Dietrich attributed this effect to “compressed” processing of entities, such that entities would be processed almost simultaneously in this task, whereas they would have been processed serially when participants had more processing time. Similarly, they argued that the interaction between discourse salience and animacy found by Prat-Sala and Branigan (2000) [i.e., context may overrule effects of (lexical) animacy] could be due to the fact that information about both factors was already present before participants started speaking, also leading to compressed processing. However, while in our experiments all information about the referents was also given before the participants started speaking, we did not find an interaction between target and competitor animacy (see footnote 2). In addition, while we did not present any linguistic context before the participants started speaking, we did find evidence that discourse context overruled animacy effects. More research is needed to investigate how choices in language production are affected by the nature of the task.

A second implication of our findings is that the choice of referring expression can be influenced by non-linguistic, perceptual information. This is unexpected under an account in which the choice of referring expression is only determined by local discourse factors, such as givenness, grammatical function, and topichood (e.g., Ariel, 1990; Gundel et al., 1993; Grosz et al., 1995; Stevenson, 2002; Kehler et al., 2008). As also found by Fukumura and van Gompel (2011), the inherent accessibility of referents can influence the choice of referring expression. Our results extend this finding by showing that it is not just an effect of conceptual representations associated with lexical items. Rather, it involves a level of non-linguistic representation (e.g., Bock and Warren, 1985; Arnold, 2010), which may be shaped by perceptual properties, such as an object’s movements. This also means that although animacy effects have been attributed to inherent accessibility, i.e., accessibility based on intrinsic properties of a (lexical) concept, these effects are also dependent on the (perceptual) context, and hence are partly driven by derived accessibility. It remains an open question whether such non-linguistic, perception-based representations should always be activated in linguistic tasks, or primarily play a role in cases of violations of canonical animacy such as in the present study. However, there is evidence that they are relevant also in more “everyday” language use (see Rosenbach, 2008 for an overview). In addition, our finding that even lexically animate entities may be treated as less animate in reference when their movements appear less animate might suggest that our results are not just due to the participants’ familiarity with cartoons or fairy tales, in which this kind of animacy shift is rare.

Combining our two main findings, a pattern emerges that seems contradictory: whereas a referent’s perceptual animacy can override lexical animacy in determining the choice of referring expression, this does not happen in the choice of referent for the subject position. This is not in line with accounts of referent accessibility in which the choice of referent for first mention and the choice of referring expression are both dependent on the conceptual accessibility of mental representations (e.g., Arnold, 2008, 2010). For example, in her Expectancy Hypothesis, Arnold (2008) proposes that accessibility is a catchall term for different factors that correlate with the probability that an entity will be mentioned again. However, the present results suggest that the different cues for animacy cannot easily be gathered under this single term. Other researchers have suggested that choice of referring expression and choice of referent for first mention should be dissociated (e.g., Stevenson et al., 1994; Kehler et al., 2008). Typically, a distinction is made between bottom-up factors, such as grammatical function and information structure, and top-down factors, such as coherence, discourse topicality, and general expectations about what will happen next. The choice of referring expression is assumed to be affected by bottom-up factors, similar to Centering (Gordon et al., 1993; Grosz et al., 1995), while the likelihood that an entity will be mentioned next is affected by top-down factors. However, our finding that perceptual animacy influences the rate of pronominalization might also seem hard to reconcile with this account. Perceptual animacy can be regarded as a top-down factor, since it is dependent on context rather than linguistic properties. By analogy with other top-down factors such as coherence, perceptual animacy should be expected to affect likelihood of next mention, but not choice of referring expression.

An explanation for the apparent contradiction may lie in the task-dependent effects outlined above. While the choice of referent and the choice of referring expression may both be influenced by the referent’s extra-linguistic conceptual accessibility, in the case of choosing a referent for the subject position this process may receive competition from the animacy of the lexical items when these are already given. That is, the presentation of a lexical item activates the corresponding lexical concept, which may boost the speed of retrieval of the lexical item in production (e.g., Bock and Irwin, 1980). This boost may be larger in the case of an animate concept. Hence, the quick retrieval of animate lexical items may make them more likely to be placed in subject position, even when the referent is conceptualized as inanimate due to the perceptual context. The choice of referring expression, on the other hand, is less dependent on the speed of lexical retrieval. Although it has been found that lexical information from the antecedent noun is activated when retrieving a pronoun (e.g., Meyer and Bock, 1999; Schmitt et al., 1999), the choice of whether or not to use a pronoun in the first place can probably be made directly on the basis of the non-linguistic conceptual representation of an entity. Hence, this choice may be more driven by accessibility derived from perceptual cues than the choice of referent.

However, since we investigated the number of subject references, and not first mention *per se*, we cannot exclude the possibility that perceptual animacy does have an effect on linearization independently of grammatical function, as has been suggested by several studies (e.g., Branigan and Feleki, 1999; Prat-Sala and Branigan, 2000; Christianson and Ferreira, 2005; Branigan et al., 2008). Whereas Dutch, the language of our experiments, has a relatively free word order, and thus allows for the investigation of linearization independently of grammatical function, our data were not structured enough to analyze this. For example, as we were dealing with relatively spontaneous speech, most of our selected clauses were not clear-cut sentences, which makes it hard to determine where exactly sentences begin. Therefore, we did not further pursue these analyses, and we will leave this issue to future research.

Still, it is not clear how our finding that perceptual animacy affects rate of pronominalization would be accounted for in models of language production. It could be argued that this effect is mediated by other factors, such as information structure or agency. For example, animate entities may be more likely to be topics (e.g., Givón, 1983). Hence, it might be the case that perceptually animate entities are more likely to be pronominalized because they are topics. This explanation would be in line with a Centering-type account. However, this is not a likely explanation. Firstly, we have seen that the effects of perceptual animacy remained intact when we controlled for discourse salience. Although entities that had been mentioned in the previous clause were likely to be pronominalized, entities that had not been mentioned (and thus were not likely topics) were more likely to be pronominalized when they were perceptually animate. Secondly, the target referent was always presented just before each animation, and it was also the first entity to appear in the screen at the start of each animation. We assume that this made all target referents equally likely candidates for the discourse topic across the conditions.

As for agency, this is another factor that is likely to affect a referent’s conceptual accessibility (e.g., Van Nice and Dietrich, 2003b), but it is hard to disentangle it from animacy, since agents are often animate. In our experiments, we kept the agency of the objects constant in the sense that it was always the target figure that moved in the episode of interest (the transitive action), while the competitors remained still. Thus, although the mere fact that the objects moved might already have increased their conceptual accessibility, this was at least the same for both the animate and the inanimate moving objects. However, a valid argument against this is that agency is itself a gradient notion (e.g., Dowty, 1991). That is, the inanimate movements in our experiments may have been inherently less agentive than the animate movements. Dowty (1991) proposed an entailment hierarchy of agentivity, in which entities are considered more agentive the more properties of a prototypical agent they possess. The hierarchy is headed by the proto-agent properties volition (i.e., having a will) and sentience (i.e., being conscious, being able to perceive), both of which entail animacy. On this view, we cannot exclude the possibility that our perceptually animate objects were also more prototypical agents than our perceptually inanimate objects. Since it would be difficult to completely disentangle perceptual animacy from perceptual agency by using motion alone, future studies could find different ways of manipulating perceptual animacy to tease these two factors apart.

These issues notwithstanding, the present study has shown that referential choices in Dutch can be influenced by factors that go beyond the linguistic context, and may even be perceptual in nature. We have focused on the interaction between two sources of animacy, and although many studies have shown that animacy is not reducible to an epiphenomenon of some other accessibility-related factor, it is clear that it interacts with many other factors (e.g., Comrie, 1989; Van Nice and Dietrich, 2003a; Rosenbach, 2008). Future research should investigate more interactions between accessibility-related factors in referential choices, especially between linguistic and perceptual factors, and between discourse-related and referent-intrinsic factors, possibly also using more online measures such as eye movements.

In summary, the present study provides evidence for differential effects of perceptual and lexical cues for animacy on referential choices. Perceptual animacy appeared to overrule lexical animacy in the choice of referring expression, extending previous findings. On the other hand, the choice of referent for the subject position appeared to be affected only by lexical animacy. The results raise new questions about the nature of animacy effects on referential choices in particular and of accessibility in general.

## Conflict of Interest Statement

The authors declare that the research was conducted in the absence of any commercial or financial relationships that could be construed as a potential conflict of interest.

## Supplementary Material

The Supplementary Material for this article can be found online at http://www.frontiersin.org/Language_Sciences/10.3389/fpsyg.2013.00154/abstract

## Footnotes

^1^One reason why such cases are hard to find may be that there are more linguistic constructions that require animate arguments than there are constructions that only take inanimate arguments. An example might be the use of animate nouns in constructions that normally only allow mass nouns, as in “That’s a lot of dog you’ve got there” (Croft, 1994).

^2^It is conceivable that the effect of lexical animacy is due to relative animacy of the target and competitors rather than target animacy alone. Since participants were free to refer to the competitor objects in any way they wanted, we did not manipulate competitor animacy systematically. However, to check whether relative animacy could have affected our results, we coded whether participants used animate or inanimate lexical items to refer to the competitor objects. We omitted an additional 10 observations in which the animacy of the competitors was unclear. Adding competitor animacy as a factor to the model revealed a main effect of competitor animacy: the target was less likely to be mentioned as the subject when the competitors were lexically animate (72.7%; *n* = 150) than when they were lexically inanimate (94.4%; *n* = 248), β = −2.28, SE = 0.42, *p* < 0.001. The main effect of target lexical animacy remained, β = 1.43, SE = 0.46, *p* < 0.01, and target perceptual animacy remained non-significant, β = 0.33, SE = 0.48, *p* = 0.50. There were no interactions between target and competitor animacy, suggesting that target animacy affects mention as subject independently from competitor animacy.

^3^Although the grammatical function of the referent in the previous sentence is an important factor in determining whether it will be the subject of the next sentence (e.g., Grosz et al., 1995), we did not distinguish between different grammatical functions here, since there were only a few cases in which the referent was mentioned as something else than the subject (3.7%). Repeating the analysis using a distinction between subject references and everything else yielded similar results.

^4^Again, we conducted an analysis in which we included competitor animacy in the model. The effect of competitor animacy was marginally significant, β = −0.53, SE = 0.32, *p* = 0.09, with slightly fewer pronominalized target referents when the competitors were lexically animate (80.7%; *n* = 150) than lexically inanimate (83.5%; *n* = 248). The significant effect of target lexical animacy remained, β = 0.71, SE = 0.29, *p* < 0.05, as did the marginally significant effect of target perceptual animacy, β = 0.53, SE = 0.30, *p* = 0.07. There were no interactions between target and competitor animacy. This suggests that target lexical animacy affects pronoun use independently from competitor lexical animacy.

^5^These examples, as well as those in the next paragraph, were constructed for illustrative purposes and do not reflect any participant’s exact wordings.

^6^See http://www.qconferencing.eu. Communication through Eye Catchers resembles face-to-face communication, because users can have direct eye contact (cf. Mol et al., 2011). Any effects of communication medium should not influence the results, since the use of Eye Catchers was counterbalanced across the animate and inanimate conditions. In addition, participants did see each other in person before the experiment and were experienced in using Eye Catchers due to the unrelated experiment that directly preceded the presently discussed experiment. Removing the four participants that communicated face-to-face from the analysis did not change the results.

^7^Although it was not the aim of this experiment to also investigate effects of discourse salience, we did perform separate analyses on the two types of context (discourse salient: referent was mentioned in the directly preceding sentence; *n* = 75, discourse non-salient: referent was not mentioned in the directly preceding sentence; *n* = 39). However, because the number of observations in each set was too small to support a mixed model with a full random effects structure, we performed two repeated measurement ANOVAs on arc-sine transformed proportions of subject references. These analyses showed no effects of perceptual animacy in either data set: discourse salient, *F*1(1,14) = 1.00, *p* = 0.33; *F*2(1,7) = 1.00, *p* = 0.35; discourse non-salient, *F*1(1,10) = 0.51, *p* = 0.49; *F*2(1,6) = 0.57, *p* = 0.48.

^8^Separate analyses were performed for discourse salient referents (*n* = 75) and discourse non-salient referents (*n* = 39). Repeated measurement ANOVAs (see footnote 7) showed a marginally significant effect of perceptual animacy in discourse salient contexts, *F*1(1,14) = 4.53, *p* = 0.05; *F*2(1,7) = 3.80, *p* = 0.09. In discourse non-salient contexts, the effect of perceptual animacy was significant but only over participants, *F*1(1,10) = 7.34, *p* < 0.05; *F*2(1,6) = 0.20, *p* = 0.67. In addition, we investigated whether the effect of animacy held for referring expressions in both subject and non-subject position. However, there were very few instances of non-subject expressions referring to the target figure (*n* = 6). When we omitted these cases from the analysis, the effect of perceptual animacy remained significant, β = 1.09, SE = 0.53, *p* < 0.05.

## Appendix

**Table A1:** **Lexical items used in Experiment 1**.

Animate		Inanimate	
schaatser	“skater”	handtas	“handbag”
prins	“prince”	voetbal	“football”
peuter	“toddler”	schoen	“shoe”
visser	“fisherman”	fles	“bottle”
padvinder	“boy scout”	steen	“stone”
koningin	“queen”	sneeuwvlok	“snowflake”
danseres	“(female) dancer”	lamp	“lamp”
boef	“scoundrel”	eierdoos	“egg carton”

**Table A2:** **Nonsense words used in Experiment 2**.

daptinder
knurp
kilper
vopper
pundimper
sloeiweurd
krielf
etalbuns

## References

[B1] Anderson G. D. S. (1997). On “animacy maximization” in Fox (Mesquakie). Int. J. Am. Linguist. 63, 227–247 10.1086/466320

[B2] Ariel M. (1990). Accessing Noun-phrase Antecedents. London: Routledge

[B3] Arnold J. E. (2008). Reference production: production-internal and addressee-oriented processes. Lang. Cogn. Process. 23, 495–527 10.1080/01690960801920099

[B4] Arnold J. E. (2010). How speakers refer: the role of accessibility. Lang. Linguist. Compass 4, 187–203 10.1111/j.1749-818X.2010.00193.x

[B5] Bock J. K. Irwin D. E. (1980). Syntactic effects of information availability in sentence production. J. Verbal Learning Verbal Behav. 19, 467–484 10.1016/S0022-5371(80)90321-7

[B6] Bock J. K. Loebell H. Morey R. (1992). From conceptual roles to structural relations: bridging the syntactic cleft. Psychol. Rev. 99, 150–171 10.1037/0033-295X.99.1.150 1546115

[B7] Bock J. K. Warren R. K. (1985). Conceptual accessibility and syntactic structure in sentence formulation. Cognition 21, 47–67 10.1016/0010-0277(85)90023-X 4075761

[B8] Boroditsky L. Schmidt L. A. Phillips W. (2003). “Sex, syntax, and semantics,” in Language in Mind: Advances in the Study of Language and Thought, eds Gentner D. Goldin-Meadow S. (Cambridge, MA: MIT Press), 61–79

[B9] Branigan H. P. Feleki E. (1999). “Conceptual accessibility and serial order in Greek speech production,” in Proceedings of the Twenty First Annual Conference of the Cognitive Science Society, (Vancouver).

[B10] Branigan H. P. Pickering M. J. Tanaka M. (2008). Contributions of animacy to grammatical function assignment and word order during production. Lingua 118, 172–189 10.1016/j.lingua.2007.02.003

[B11] Christianson K. Ferreira F. (2005). Conceptual accessibility and sentence production in a free word order language (Odawa). Cognition 98, 105–135 10.1016/j.cognition.2004.10.006 16307955

[B12] Comrie B. (1989). Language Universals and Linguistic Typology, 2nd Edn. Chicago: University of Chicago Press

[B13] Croft W. (1994). “Voice: beyond control and affectedness,” in Voice: Form and Function, eds Fox B. A. Hopper P. J. (Amsterdam: John Benjamins), 89–118

[B14] Dabrowska E. (1998). How metaphor affects grammatical coding: the Saxon genitive in computer manuals. Engl. Lang. Linguist. 2, 121–127

[B15] Dahl Ö (2000). “Animacy and the notion of semantic gender,” in Gender in Grammar and Cognition, eds Unterbeck B. Rissanen M. (Berlin: Mouton de Gruyter), 99–115

[B16] Dahl Ö (2008). Animacy and egophoricity: grammar, ontology and phylogeny. Lingua 118, 141–150 10.1016/j.lingua.2007.02.008

[B17] Dahl Ö. Fraurud K. (1996). “Animacy in grammar and discourse,” in Reference and Referent Accessibility, eds Fretheim T. Gundel J. (Amsterdam: John Benjamins), 47–64

[B18] Dowty D. (1991). Thematic proto-roles and argument selection. Language 67, 547–619 10.2307/415037

[B19] Ferreira V. S. Yoshita H. (2003). Given-new ordering effects on the production of scrambled sentences in Japanese. J. Psycholinguist. Res. 32, 669–692 10.1023/A:1026146332132 14653013

[B20] Fraurud K. (1996). “Cognitive ontology and NP form,” in Reference and Referent Accessibility, eds Fretheim T. Gundel J. K. (Amsterdam: John Benjamins), 65–87

[B21] Fukumura K. van Gompel R. P. G. (2011). The effect of animacy on the choice of referring expression. Lang. Cogn. Process. 26, 1472–1504 10.1080/01690965.2010.506444

[B22] Gelman R. Durgin F. Kaufman L. (1995). “Distinguishing between animates and inanimates: not by motion alone,” in Causal Cognition: A Multidisciplinary Debate, eds Sperber D. Premack D. Premack A. J. (Oxford: Clarendon Press), 150–184

[B23] Givón T. (1983). Topic Continuity in Discourse. Amsterdam: John Benjamins

[B24] Gordon P. C. Grosz B. J. Gilliom L. A. (1993). Pronouns, names and the centering of attention in discourse. Cogn. Sci. 7, 311–347 10.1207/s15516709cog1703_1

[B25] Grosz B. J. Joshi A. K. Weinstein S. (1995). Centering: a framework for modelling the local coherence of discourse. Comput. Linguist. 21, 203–225

[B26] Gundel J. K. Hedberg N. Zacharski R. (1993). Cognitive status and the form of referring expressions in discourse. Language 69, 274–307 10.2307/416535

[B27] Heider F. Simmel M. (1944). An experimental study of apparent behavior. Am. J. Psychol. 57, 243–259 10.2307/1416950

[B28] Jaeger F. (2008). Categorical data analysis: away from ANOVAs (transformation or not) and towards logit mixed models. J. Mem. Lang. 59, 434–446 10.1016/j.jml.2007.11.007 19884961 PMC2613284

[B29] Katz J. (1972). Semantic Theory. New York: Harper & Row, Publishers

[B30] Kehler A. Kertz L. Rohde H. Elman J. L. (2008). Coherence and coreference revisited. J. Semant. 25, 1–44 10.1093/jos/ffm018 22923856 PMC3424618

[B31] Kuno S. Kaburaki E. (1977). Empathy and syntax. Linguist. Inq. 8, 627–672

[B32] Lakoff G. Johnson M. (1980). Metaphors We Live By. Chicago: University of Chicago Press

[B33] Levelt W. J. M. Roelofs A. Meyer A. S. (1999). A theory of lexical access in speech production. Behav. Brain Sci. 22, 1–75 10.1017/S0140525X99451775 11301520

[B34] Maes A. A. (1997). Referent ontology and centering in discourse. J. Semant. 14, 207–235 10.1093/jos/14.3.207

[B35] McDonald J. Bock J. K. Kelly M. H. (1993). Word and World order: semantic, phonological and metrical determinants of serial position. Cogn. Psychol. 25, 188–230 10.1006/cogp.1993.1005 8482072

[B36] Meyer A. S. Bock J. K. (1999). Representations and processes in the production of pronouns: some perspectives from Dutch. J. Mem. Lang. 41, 281–301 10.1006/jmla.1999.2649

[B37] Mol E. M. M. Krahmer E. J. Maes A. A. Swerts M. G. J. (2011). Seeing and being seen: the effects on gesture production. J. Comput. Mediat. Commun. 17, 77–100 10.1111/j.1083-6101.2011.01558.x

[B38] Nieuwland M. S. Van Berkum J. J. A. (2006). When peanuts fall in love: N400 evidence for the power of discourse. J. Cogn. Neurosci. 18, 1098–1111 10.1162/jocn.2006.18.7.1098 16839284

[B39] Nunberg G. (1979). The non-uniqueness of semantic solutions: polysemy. Linguist. Philos. 3, 143–184 10.1007/BF00126509

[B40] Prat-Sala M. Branigan H. P. (2000). Discourse constraints on syntactic processing in language production: a cross-linguistic study in English and Spanish. J. Mem. Lang. 42, 168–182 10.1006/jmla.1999.2668

[B41] Rosenbach A. (2008). Animacy and grammatical variation – findings from English genitive variation. Lingua 118, 151–171 10.1016/j.lingua.2007.02.002

[B42] Santos N. S. David N. Bente G. Vogeley K. (2008). Parametric induction of animacy experience. Conscious. Cogn. 17, 425–437 10.1016/j.concog.2008.03.012 18440830

[B43] Schmitt B. M. Meyer A. S. Levelt W. J. M. (1999). Lexical access in the production of pronouns. Cognition 69, 313–335 10.1016/S0010-0277(98)00073-0 10193050

[B44] Scholl B. J. Tremoulet P. D. (2000). Perceptual causality and animacy. Trends Cogn. Sci. (Regul. Ed.) 4, 299–309 10.1016/S1364-6613(00)01506-0 10904254

[B45] Schultz J. Friston K. J. O’Doherty J. Wolpert D. M. Frith C. D. (2005). Activation in posterior superior temporal sulcus parallels parameter inducing the percept of animacy. Neuron 45, 625–635 10.1016/j.neuron.2004.12.052 15721247

[B46] Sridhar S. N. (1988). Cognition and Sentence Production: A Cross-linguistic Study. New York: Springer-Verlag

[B47] Stevenson R. J. Crawley R. A. Kleinman D. (1994). Thematic roles, focus and the representation of events. Lang. Cogn. Process. 94, 473–592

[B48] Stevenson R. J. (2002). “The Role of Salience in the Production of Referring Expressions,” in Information Sharing: Reference and Presupposition in Language Generation and Interpretation, eds van Deemter K. Kibble R. (Stanford, CA: CSLI Publications), 167–192

[B49] Tremoulet P. D. Feldman J. (2000). Perception of animacy from the motion of a single object. Perception 29, 943–951 10.1068/p3101 11145086

[B50] Van Nice K. Y. Dietrich R. (2003a). “Animacy effects in language production: from mental model to formulator,” in Mediating Between Concepts and Grammar, eds Härtl H. Tappe H. (Berlin: Mouton de Gruyter), 101–117

[B51] Van Nice K. Y. Dietrich R. (2003b). Task sensitivity of animacy effects: evidence from German picture descriptions. Linguistics 41, 825–849

[B52] Wiese H. (2003). “Semantics as a gateway to language,” in Mediating Between Concepts and Grammar, eds Härtl H. Tappe H. (Berlin: Mouton de Gruyter), 197–222

[B53] Yamamoto M. (1999). Animacy and Reference: A Cognitive Approach to Corpus Linguistics. Amsterdam: John Benjamins

